# Associations of *TP53* codon 72 polymorphism with complications and comorbidities in patients with type 1 diabetes

**DOI:** 10.1007/s00109-020-02035-1

**Published:** 2021-01-25

**Authors:** Bartosz Słomiński, Maria Skrzypkowska, Monika Ryba-Stanisławowska, Małgorzata Myśliwiec, Piotr Trzonkowski

**Affiliations:** 1 Department of Medical Immunology, Faculty of Medicine, Medical University of Gdańsk, ul. Dębinki 1, 80-211 Gdańsk, Poland; 2 Chair & Clinics of Paediatrics, Diabetology and Endocrinology, Faculty of Medicine, Medical University of Gdańsk, Dębinki 7, 80-211 Gdańsk, Poland

**Keywords:** Type 1 diabetes, Diabetes complications, *TP53* codon 72 polymorphism

## Abstract

**Abstract:**

Wild-type TP53 plays an important role in the regulation of immune response and systemic inflammation. In type 1 diabetes (T1D), *TP53* pathways are upregulated and an increased susceptibility to apoptosis is observed. We hypothesize that *TP53* codon 72 polymorphism could be associated with complications and comorbidities in patients with T1D. We have investigated the associations of the *TP53* codon 72 polymorphism with the T1D complications and comorbidities (retinopathy, nephropathy, hypertension, dyslipidemia, autoimmune thyroiditis, and celiac disease) in 350 patients. The key results of our approach are as follows: (1) In diabetic subjects, the Pro/Pro genotype is associated with an increased risk of microvascular complications, dyslipidemia, and celiac disease; (2) the Arg/Arg variant is associated with a decreased risk of autoimmune thyroiditis and celiac disease; (3) the Pro allele is associated with an increased risk of dyslipidemia, autoimmune thyroiditis, and celiac disease. Although further studies are required, our results for the first time indicate that the *TP53* codon 72 polymorphism could be considered a genetic marker to predict the increased susceptibility to some T1D complications and comorbidities.

**Key messages:**

- We analyzed the *TP53* codon 72 polymorphism in patients with T1D.
- Pro/Pro genotype is associated with an increased risk of microvascular complications, dyslipidemia, and celiac disease.
- The Arg/Arg variant is associated with a decreased risk of autoimmune thyroiditis and celiac disease.
- The Pro allele is associated with an increased risk of dyslipidemia, autoimmune thyroiditis, and celiac disease.

## Introduction

Wild-type TP53 (tumor protein p53, more commonly known as P53) has been established as a tumor suppressor in human cancer as it plays an important role in the control of cell proliferation and death. P53 is a transcription factor that protects the genome against a plethora of environmental and intracellular challenges [1]. The P53 protein is activated when DNA damage occurs by stress such as ultraviolet radiation, heat shock, growth factor withdrawal, hypoxia, and inflammation in various cells and tissues [2]. These stresses have an impact upon many tissue and organ functions and therefore can lead to many diverse disorders or even regulate normal organismic functions. The consequence of P53 positive regulation is the induction of pathways leading to cell cycle arrest, apoptosis, DNA repair, autophagy, and senescence [3]. Not only is P53 activated by stress signals, but it also seems to control energy metabolism under normal conditions [4]. P53 has been also found to be a critical factor governing immune responses and inflammation, aging, reproduction, development, and neurodegeneration [5]. A number of studies suggest that P53 plays a protective role against various autoimmune conditions by suppressing cytokine production as well as reducing the number of pathogenic cells [6].

*TP53* gene presents a common polymorphism at codon 72 of exon 4 (rs1042522) characterized by the substitution of cytosine (C) by a guanine (G) that confers a change of ancestral proline to arginine in the amino acid sequence. The frequency of the Pro allele ranges from 70% among South Africans to 23% among Western Europeans. Pro is probably the ancient allele, but the reason for the high frequency of Arg among Europeans is unclear [7]. The two resulting variants (Pro and Arg) are neither biochemically nor biologically equivalent [8]. At the cellular level, the Arg variant is a stronger apoptosis inducer while the Pro variant is a more powerful transcriptional activator that induces a higher level of cell cycle arrest [9]. Therefore, a large number of studies have explored the role of *TP53* codon 72 polymorphism in cancer providing mixed and confusing results. Less is known about the relations between the *TP53* codon 72 polymorphism and other clinical conditions.

Considering increasingly appreciated role of *P53* in the regulation of immune response and systemic inflammation, we were interested whether common functional *TP53* polymorphism may affect diabetes complications and comorbidities.

## Materials and methods

### Subjects

This study was conducted with 350 Caucasoid adolescents, including 171 boys and 179 girls (mean age 15.5 ± 3.5 years) with clinical and laboratory diagnosis of T1D, recruited from the Chair and Clinics of Pediatrics, Diabetology and Endocrinology, Medical University of Gdańsk. T1D diagnosis was based on the American Diabetes Association criteria [10]. All patients were treated with humanized insulin at doses of 0.87 ± 0.2 U/kg. At the time of sampling, lipid levels (total cholesterol – TC, triglycerides – TG, high-density lipoprotein cholesterol – HDL, low-density lipoprotein cholesterol – LDL) along with biochemical measurement of renal function, C-reactive protein (CRP), and glycated hemoglobin (HbA1c) were monitored. All of the subjects with diabetes-related complications and comorbidities were newly diagnosed and previously untreated.

Population control subjects consisted of 200 healthy volunteers from the same population. Neither signs of autoimmune and/or inflammatory disease at the time of sampling nor evidence of T1D in families were disclosed as confirmed by medical records and laboratory tests.

Written informed consent to participate in the study was obtained from all subjects or from their parents. This study was approved by the Ethics Committee of the Medical University of Gdańsk (NKEBN/2014/2009; 2009) and the investigation was carried out in accordance with the principles of the Declaration of Helsinki.

### Medical examinations

Systolic and diastolic blood pressures (SBP and DBP, resp.) were measured using automatic 24-h ambulatory blood pressure monitoring (ABPM) by the Holter method. All the average values of the blood pressure were expressed in the centyle charts. Arterial hypertension was diagnosed when the blood pressure value reached at least the 95th percentile for the corresponding age, gender, and height on at least three separate occasions [11].

Ophthalmologic investigation was performed in all TD1 patients. Diabetic retinopathy was determined by visual acuity, intraocular pressure measurement, anterior segment estimation by slit lamp (TOPCON SL-82, Japan), and fluorescein angiography (digital camera-Topcon IMAGEnet2000, Japan). The eye fundus examination was performed with the + 90D lens (Ocular Instruments Inc., Bellevue, WA, USA). Each image was graded for retinopathy according to the Early Treatment for Diabetic Retinopathy Study (ETDRS) severity level and was dichotomized as having retinopathy (level 15 and above) or not having retinopathy (≤ 14) [12].

Renal function was determined by estimated glomerular filtration rate (eGFR), which was evaluated by using the Zappitelli equation: eGFR (ml/min/1.73 m^2^) =  (507.76 _*_ e^(0.3 * height (cm))^)/(serum cystatin C (mg/l) ^0.635^_*_ serum creatinine (μmol/l) ^0.547^) [13].

The urinary albumin excretion (UAE) was expressed as the average of three 24-h collections. Cases were classified as microalbuminuria when in at least two out of three urine samples, UAE ratio was > 30–300 mg/24 h. Diabetic nephropathy was defined as persistent microalbuminuria in two out of three consecutive urine samples without clinical or laboratory evidence of other kidney or urinary tract disease.

Dyslipidemia was defined by the presence of one or more abnormal serum lipid concentrations: TC ≥ 5.17 mmol/l (200 mg/dl); HDL < 1.03 mmol/l (40 mg/dl); LDL ≥ 2.6 mmol/l (100 mg/dl); TG ≥ 1.69 mmol/l (150 mg/dl) [14]. Further analyses were performed after controlling for age and pubertal stage to avoid differences in lipid values [15].

In all of the patients, the diagnosis of celiac disease (CD) was made in accordance with the revised criteria of the European Society for Pediatric Gastroenterology, Hepatology, and Nutrition [16]. To be defined as celiac sufferer, each subject was required to have (1) positive celiac-specific antibodies (IgA-AGA/IgG-AGA; IgA-EmA/IgG-EmA and IgA-anti-tTG); (2) or a proximal small intestinal biopsy compatible with celiac disease; and (3) either clinical and/or histological improvement with a gluten-free diet. Celiac patients fulfilled all three criteria.

Screening for autoimmune thyroiditis (TA) was performed using measurements of thyroid antiperoxidase antibody (TPOAb), antithyroglobulin antibody (TGAb), and thyroid-stimulating hormone (TSH) receptor antibodies (TSHRAb) and sonographic signs of the disease [17]. Free thyroxine and TSH were also measured. TA was defined as the presence of at least one thyroid autoantibody.

### Methods

Venous blood samples were withdrawn after 12–14 h overnight fasting. Serum and plasma samples were collected from T1D patients by centrifugation at 500*g* for 15 min and stored at − 70 °C until analysis.

Concentrations of TNF-α, ICAM-1, VCAM-1, IL-6, and IL-10 were determined using commercial enzyme-linked immunosorbent assay kits (R&D Systems, Minneapolis, MN, USA) according to the manufacturer’s protocol.

Plasma TC, TG, and HDL-C concentrations were measured in an independent, ISO-certified laboratory. LDL-C was estimated by the Friedewald equation [18].

### Genotyping protocol

Genomic DNA from all subjects was isolated from EDTA-stabilized blood using the EXTRACTME DNA BLOOD kit (Blirt, Poland). DNA was stored at − 20 °C until the time of use.

The genotyping of *TP53* codon 72 polymorphism (rs1042522) was carried out using tetra-primer amplification refractory mutation system–polymerase chain reaction (ARMS–PCR). In this assay, confronting pairs of primers (outers and inners) were used as shown below:

- *forward outer*: 5′ – ACAAGGGTTGGGCTGGGGACCTGGAGGG – 3′.
- *reverse outer*: 5′ – CAGCCCCTCAGGGCAACTGACCGTGCAA – 3′.
- *forward inner*: 5′ – CTCCCAGAATGCCAGAGGCTGCTCCGCC – 3′.
- *reverse inner*: 5′ – GTAGGAGCTGCTGGTGCAGGGGCCAGGC – 3’.

The region containing *TP53* codon 72 polymorphism was amplified in a total volume of 15 μl, containing 20 ng of DNA template, 1.65 mM MgCl_2_ (Thermo Fisher Scientific, MA, USA), 200 μM dNTP (Thermo Fisher Scientific, MA, USA), 250 nM of each primer (Sigma-Aldrich, MO, USA), and 0.75 U FIREPol DNA polymerase with 1x buffer (Solis BioDyne, Tartu, Estonia). The procedure consisted of denaturation at 96 °C for 15 min, followed by 35 cycles of 96 °C for 30 s, 68 °C for 30 s, 72 °C for 30 s, and a final extension at 72 °C for 1 min. PCR products were visualized on a 2% agarose gel with ethidium bromide staining. Genotyping was performed as follows: 403, 249 bp for Arg/Arg (GG) genotype; 403, 249, 210 bp for Arg/Pro (GC) genotype; and 403, 210 bp for Pro/Pro (CC) genotype.

DNA samples were first sequenced to establish three *TP53* gene polymorphic variants as a quality control. Afterwards, DNA samples of the Arg/Arg, Arg/Pro, and Pro/Pro individuals were routinely added to the examined ones to ensure genotype accuracy.

### Statistical analysis

The results were analyzed using Statistica, ver. 12 (StatSoft, Inc., USA). Conformation of the allele frequencies to the Hardy-Weinberg equilibrium (HWE) proportions was tested by the *χ*^2^ test. The genotypes and allele frequencies of the *TP53* codon 72 polymorphism were compared using Pearson’s *χ*^2^ test. Differences between groups were analyzed by ANOVA for normally distributed values or the Kruskal–Wallis test for nonparametric values (the post hoc NIR test was applied to assess statistical significance) and by the *χ*^2^ Pearson test for dichotomous variables. Correlation between variables was evaluated using Spearman’s correlation coefficient. To deal with multiple testing, Benjamini-Hochberg’s correction was used for statistical significance. The level of significance was set at *p* ≤ 0.05. Logistic regression model was used to examine the association between *TP53* codon 72 polymorphism and diabetes-related complications and comorbidities.

## Results

### TP53 codon 72 genotype distribution

*TP53* codon 72 genotypes were analyzed in T1D patients and healthy controls. The occurrence of each genotype and allele frequencies is shown in Table 1. The genotype distributions for both, healthy group and T1D patients, were in Hardy-Weinberg equilibrium (*p* = 0.73 and 0.69, resp.). Comparison of the frequencies of *TP53* codon 72 genotypes between healthy group and the T1D patients revealed lack of significant differences (*p* = 0.11) but the presence of Arg/Pro variant was connected with a some increased risk of T1D (OR = 1.457, *p* = 0.04). In case of the allele frequencies among both groups, no differences were found (*p* = 0.15).

**Table 1:** Distribution of genotype and allele frequencies of *TP53* codon 72 polymorphism in healthy group and patients with T1D

*TP53* genotypes	Healthy (*N* = 200)		T1D (*N* = 350)		*χ*^2^ Pearson	Odds ratio analysis		
	*N*	%	*N*	%	*p*	OR	95% CI	*p*
Arg/Arg	112	56.0	166	47.4	*χ*^2^ = 4.39 *p* = 0.11	0.709	0.499–1.006	0.05
Arg/Pro	69	34.5	152	43.4		1.457	1.101–2.091	**0.04**
Pro/Pro	19	9.5	32	9.2		0.958	0.531–1.731	0.89
Allele frequency								
Arg	293	73.3	484	69.2	*χ*^2^ = 2.07 *p* = 0.15	0.818	0.622–1.076	0.15
Pro	107	26.7	216	30.8		1.222	0.929–1.607	

Bold *p* values indicate that the differences are statistically significant

*N* number of patients, *OR* odds ratio, *95% CI* 95% confidence interval

### TP53 codon 72 polymorphism and clinical characteristics of patients

Characteristics of T1D patients included in this study differing in the *TP53* codon 72 polymorphism are shown in Table 2. There were no statistically significant differences in sex, age, age of T1D onset, duration of T1D, BMI, HbA1c, and values of blood pressure between subjects with different *TP53* genotypes. However, individuals with Pro/Pro variant had lowest values of eGFR (*p* = 0.02). There were no statistically significant differences in all clinical parameters between Arg and Pro alleles.

**Table 2:** Selected clinical characteristics of T1D patients stratified according to *TP53* codon 72 genotypes and alleles

Clinical parameter	*TP53* genotypes			*p*^1^	*p*^2^	*p*^3^	*p*^4^	*TP53* alleles		*p*^5^
	Arg/Arg	Arg/Pro	Pro/Pro					Arg	Pro	
*N* (%)	166	152	32	-	-	-	-	484	216	-
Sex (male/female)	81/85	79/73	11/21	0.19	-	-	-	241/243	101/115	0.46
Age (years)	15.6 ± 3.4	15.3 ± 3.1	16.5 ± 3.2	0.14	0.31	0.17	0.05	15.6 ± 3.2	15.5 ± 3.3	0.67
Age of onset of diabetes (years)	8.9 ± 3.1	8.3 ± 3.1	9.1 ± 2.8	0.20	0.12	0.65	0.18	8.6 ± 3.1	8.7 ± 3.1	0.61
Duration of diabetes (years)	6.8 ± 2.8	6.9 ± 2.8	7.6 ± 3.5	0.35	0.69	0.15	0.23	7.1 ± 3.0	6.8 ± 2.8	0.22
BMI (kg/m^2^)	20 ± 2	20 ± 2	21 ± 3	0.16	0.25	0.24	0.07	20 ± 3	20 ± 2	0.84
HbA1c (%) (mmol/mol)	8.5 ± 1.7	8.7 ± 1.6	8.6 ± 1.4	0.60	0.32	0.76	0.78	8.7 ± 1.5	8.6 ± 1.7	0.45
	70 ± 19	72 ± 17	71 ± 16					71 ± 17	70 ± 18	
eGFR (ml/min/1.73 m^2^)	121 ± 26	128 ± 26	116 ± 27	**0.02**	**0.02**	0.36	**0.02**	124 ± 27	123 ± 26	0.54
Systolic blood pressure (mmHg)	116 ± 8	115 ± 8	113 ± 8	0.28	0.61	0.11	0.19	115 ± 7	115 ± 8	0.16
Diastolic blood pressure (mmHg)	72 ± 6	73 ± 6	72 ± 5	0.76	0.55	0.79	0.55	72 ± 6	72 ± 6	0.89

Bold *p* values indicate that the differences are statistically significant

*N* number of patients, *p*^*1*^ the comparison between all genotypes, *p*^*2*^ the post hoc comparison Arg/Arg vs. Arg/Pro, *p*^*3*^ the post hoc comparison Arg/Arg vs. Pro/Pro, *p*^*4*^ the post hoc comparison Arg/Pro vs. Pro/Pro, *p*^*5*^ the comparison Arg vs. Pro

### Genotype and allele distribution of TP53 codon 72 in T1D patients considering complications and comorbidities

We have compared the distribution of genotypes and alleles between individuals with and without T1D complications and comorbidities (Table 3). None deviated significantly from HWE in all studies. There were no differences in the genotypic and allelic distributions with respect to retinopathy (*p* = 0.57 and 0.60, resp.), nephropathy (*p* = 0.26 and 0.37, resp.), and hypertension (*p* = 0.25 and 0.72, resp.). However, we have observed alterations in the frequencies of *TP53* codon 72 genotypes, but not alleles, due to diabetic microvascular complications (both retinopathy and nephropathy). Genotype distributions in patients with nephropathy and retinopathy were different in comparison to complication-free group (*p* = 0.02). We have also found differences in the genotypic and allelic distributions with respect to dyslipidemia (*p* = 0.002 and 0.03, resp.), autoimmune thyroiditis (*p* = 0.03 and 0.01, resp.), and celiac disease (*p* < 0.000 and 0.000, resp.).

**Table 3:** Genotype and allele distribution of *TP53* codon 72 polymorphism in T1D patients considering complications and comorbidities

T1D complications and comorbidities		*TP53* genotypes						*p*^1^	HWE	*TP53* alleles				*p*^2^
		Arg/Arg		Arg/Pro		Pro/Pro				Arg		Pro		
		*N*	%	*N*	%	*N*	%			*N*	%	*N*	%	
Microvascular complications	No (*N* = 225)	106	47.1	105	46.7	14	6.2	**0.02**	0.70	317	70.4	133	29.6	0.32
	Yes (*N* = 125)	60	48.0	47	37.6	18	14.4		0.67	167	66.8	83	33.2	
Retinopathy	No (*N* = 284)	136	47.6	126	44.0	24	8.4	0.57	0.69	398	69.6	174	30.4	0.60
	Yes (*N* = 64)	30	46.9	26	40.6	8	12.5		0.67	86	67.2	42	32.8	
Nephropathy	No (*N* = 270)	129	47.8	120	44.4	21	7.8	0.26	0.70	378	70.0	162	30.0	0.37
	Yes (*N* = 80)	37	46.2	32	40.0	11	13.8		0.66	106	66.3	54	33.7	
Hypertension	No (*N* = 284)	136	47.8	119	42.0	29	10.2	0.25	0.69	391	68.8	177	31.2	0.72
	Yes (*N* = 66)	30	45.5	33	50.0	3	4.5		0.70	93	70.4	39	29.6	
Dyslipidemia	No (*N* = 160)	94	49.5	88	46.3	8	4.2	**0.002**	0.73	276	72.6	104	27.4	**0.03**
	Yes (*N* = 190)	72	45.0	64	40.0	24	15.0		0.65	208	65.0	112	35.0	
Autoimmune thyroiditis	No (*N* = 265)	135	51.0	110	41.5	20	7.5	**0.03**	0.72	380	71.7	150	28.3	**0.01**
	Yes (*N* = 85)	31	36.5	42	49.4	12	14.1		0.61	104	61.2	66	38.8	
Celiac disease	No (*N* = 321)	160	49.8	137	42.7	24	7.5	**< 0.000**	0.71	457	71.2	185	28.8	**< 0.000**
	Yes (*N* = 29)	6	20.7	15	51.7	8	27.6		0.47	27	46.5	31	53.5	

Bold *p* values indicate that the differences are statistically significant

Microvascular complications = retinopathy and nephropathy

*N* number of patients, *p*^*1*^ the comparison between all genotypes, *p*^*2*^ the comparison Arg vs. Pro, *HWE* Hardy-Weinberg equilibrium

### Associations of TP53 codon 72 polymorphism with complications and comorbidities in patients with type 1 diabetes

Among the variables reported in Table 3 that were found to be significantly different between individuals with and without T1D complications and comorbidities, the logistic regression model was performed. Table 4 shows the results of *TP53* genotype and allele interaction effects for microvascular complications, dyslipidemia, autoimmune thyroiditis, and celiac disease. No significant interaction effect of genotype was observed in the other studied variables (data not shown).

**Table 4:** Odds ratio analysis for complications and comorbidities in T1D patients

T1D complications and comorbidities	*TP53* genotypes									*TP53* alleles		
	Arg/Arg			Arg/Pro			Pro/Pro			Arg^1^ vs. Pro		
	OR	95% CI	*p*	OR	95% CI	*p*	OR	95% CI	*p*	OR	95% CI	*p*
Microvascular complications	1.036	0.666–1.612	0.87	0.689	0.440–1.078	0.10	2.535	1.211–5.307	**0.01**	1.185	0.849–1.652	0.32
Dyslipidemia	0.835	0.547–1.276	0.40	0.773	0.504–1.185	0.23	4.015	1.745–9.238	**0.001**	1.429	1.035–1.973	**0.03**
Autoimmune thyroiditis	0.553	0.333–0.916	**0.02**	1.376	0.841–2.251	0.20	2.014	0.937–4.325	0.07	1.608	1.119–2.310	**0.01**
Celiac disease	0.262	0.104–0.664	**0.004**	1.439	0.670–3.089	0.35	4.714	1.883–11.801	**< 0.000**	2.836	1.645–4.888	**< 0.000**

Bold *p* values indicate that the differences are statistically significant

Microvascular complications = retinopathy and nephropathy

Arg^1^–Arg = reference allele

*OR* odds ratio, *95% CI* 95% confidence interval

Logistic regression analysis revealed a tendency of the Pro/Pro variant to associate with microvascular complications (OR = 2.535, *p* = 0.01), dyslipidemia (OR = 4.015, *p* = 0.001), and celiac disease (OR = 4.714, *p* < 0.000) where the minor genotype increases the risk of these conditions. Simultaneously, there were characteristic features linking Arg/Arg carriers and autoimmune thyroiditis (OR = 0.553, *p* = 0.02) and celiac disease (OR = 0.262, *p* = 0.004) where the major genotype decreases the risk of these conditions.

Logistic regression analysis also revealed a significant associations between Pro allele and dyslipidemia (OR = 1.429, *p* = 0.03), autoimmune thyroiditis (OR = 1.608, *p* = 0.01), and celiac disease (OR = 2.836, *p* < 0.000) with this variant increasing the risk of these conditions.

### Serum concentrations of different variables in patients with T1D differing in the TP53 codon 72 polymorphism

Table 5 describes the association between *TP53* genotypes and serum concentrations of different variables in T1D patients. There was no statistically significant difference in serum concentrations of TNF-α, IL-6, IL-10, and triglycerides between subjects with different *TP53* genotypes. However, Pro/Pro carriers had the lowest concentrations of ICAM-1 (*p* < 0.000), VCAM-1 (*p* < 0.000), and HDL-C (*p* = 0.04) and the highest levels of total cholesterol (*p* < 0.000) and LDL-C (*p* < 0.000). Simultaneously, the Arg/Arg carriers had increased serum concentrations of CRP (*p* = 0.03) and decreased IL-6/IL-10 ratio (*p* = 0.001) when compared to holders bearing other genotypes.

**Table 5:** Serum concentrations of different variables in patients with T1D differing in the *TP53* codon 72 polymorphism

Clinical parameter	*TP53* genotypes			*p*^1^	*p*^2^	*p*^3^	*p*^4^	*TP53* alleles		*p*^5^
	Arg/Arg	Arg/Pro	Pro/Pro					Arg	Pro	
TNF-α (pg/ml)	1.02 ± 0.90	1.06 ± 0.96	1.25 ± 0.85	0.44	0.68	0.20	0.30	1.03 ± 0.91	1.12 ± 0.93	0.26
CRP (mg/l)	2.17 ± 1.58	1.78 ± 1.18	1.84 ± 1.08	**0.03**	**0.01**	0.21	0.82	2.05 ± 1.48	1.80 ± 1.15	**0.03**
ICAM-1 (ng/ml)	514 ± 100	532 ± 134	439 ± 49	**< 0.00**	0.15	**< 0.00**	**< 0.00**	520 ± 112	505 ± 123	0.11
VCAM-1 (ng/ml)	812 ± 169	903 ± 196	745 ± 91	**< 0.00**	**< 0.00**	**0.04**	**< 0.00**	841 ± 183	856 ± 186	0.30
IL-6 (pg/ml)	1.45 ± 0.98	1.59 ± 1.12	1.25 ± 1.10	0.21	0.26	0.32	0.10	1.49 ± 1.03	1.49 ± 1.12	0.92
IL-10 (pg/ml)	2.09 ± 1.93	2.45 ± 2.28	2.40 ± 2.37	0.31	0.14	0.45	0.90	2.20 ± 2.05	2.43 ± 2.29	0.19
IL-6/IL-10	1.58 ± 1.04	2.30 ± 2.33	2.20 ± 1.79	**0.001**	**< 0.00**	0.08	0.78	1.81 ± 1.59	2.27 ± 2.18	**0.002**
Total cholesterol (mmol/l)	4.53 ± 0.63	4.36 ± 0.58	5.25 ± 0.69	**< 0.00**	**0.02**	**< 0.00**	**< 0.00**	4.48 ± 0.62	4.63 ± 0.73	**0.006**
HDL-C (mmol/l)	1.54 ± 0.29	1.62 ± 0.29	1.54 ± 0.15	**0.04**	**0.02**	0.92	0.14	1.57 ± 0.29	1.59 ± 0.25	0.23
LDL-C (mmol/l)	2.46 ± 0.49	2.40 ± 0.57	3.05 ± 0.75	**< 0.00**	0.39	**< 0.00**	**< 0.00**	2.44 ± 0.52	2.60 ± 0.69	**0.001**
Triglycerides (mmol/l)	1.05 ± 0.51	1.02 ± 0.39	1.13 ± 0.34	0.46	0.59	0.36	0.22	1.04 ± 0.47	1.05 ± 0.38	0.72

Bold *p* values indicate that the differences are statistically significant

*N* number of patients, *p*^*1*^ the comparison between all genotypes, *p*^*2*^ the post hoc comparison Arg/Arg vs. Arg/Pro, *p*^*3*^ the post hoc comparison Arg/Arg vs. Pro/Pro, *p*^*4*^ the post hoc comparison Arg/Pro vs. Pro/Pro, *p*^*5*^ the comparison Arg vs. Pro

There were also considerable differences between the alleles. Pro carriers had the lowest concentrations of CRP (*p* = 0.03) and total cholesterol (*p* = 0.006) and the highest of IL-6/IL-10 ratio (*p* = 0.002) and LDL-C (*p* = 0.001).

## Discussion

Linkage between *TP53* codon 72 polymorphism and type 2 diabetes (T2D) has been described in various studies. Results from the latest meta-analysis revealed that the Arg variant is one of the strongest genetic risk factors for T2D [19]. To the best of our knowledge, there were only three genetic investigations to establish the role of *TP53* codon 72 polymorphism in T1D. Spitsina et al. [20] have not found any associations, and Bitti et al. [21] have suggested that the Arg/Arg genotype predispose to T1D in a sex-specific and age-specific manner, whereas Gloria-Bottini et al. [22] have found increase in the Arg/Arg genotype in T1D vs. control subjects. In our study, T1D seemed to have little association (*p* = 0.04) with Arg/Pro carriers but not with the other genotypes nor alleles. This controversy might be due to the fact that the genotype distribution of the *TP53* codon 72 polymorphism varies among racial and ethnic groups. Interestingly, the frequency of this *TP53* variant is also associated with UV exposure, increasing latitude or colder winter temperatures [23].

In the currently available literature, there are no studies investigating the associations of *TP53* codon 72 polymorphism with T1D complications and comorbidities such as retinopathy, nephropathy, hypertension, dyslipidemia, autoimmune thyroiditis, and celiac disease. Our results indicate for the first time that this polymorphism may affect the abovementioned conditions. The key results of our approach are as follows:

1. In diabetic subjects, the Pro/Pro genotype is associated with an increased risk of microvascular complications (OR = 2.535), dyslipidemia (OR = 4.015), and celiac disease (OR = 4.714);
2. The Arg/Arg variant is associated with a decreased risk of autoimmune thyroiditis (OR = 0.553) and celiac disease (OR = 0.262);
3. The Pro allele is associated with an increased risk of dyslipidemia (OR = 1.429), autoimmune thyroiditis (OR = 1.608), and celiac disease (OR = 2.836).

There is much evidence that inflammation is an important player in the T1D complications and comorbidities. Our data imply various effects of *TP53* codon 72 polymorphism on the inflammatory status in patients. The Arg/Arg homozygotes and Arg carriers had the highest concentrations of CRP and Pro/Pro carriers had the lowest levels of proinflammatory ICAM-1 and VCAM-1. Therefore, Pro/Pro carriers seem to express weakened inflammatory response. On the other hand, individuals bearing Arg/Arg variant exhibited the lowest values of IL-6/IL-10 ratio as opposed to Pro carriers. In the light of the foregoing, patients carrying the Pro allele are more privileged.

Leaving aside our differences, accumulating evidence strongly indicates that P53 plays a significant role in the inflammation and autoimmunity [24]. The mechanisms underlying P53-mediated inflammation have been extensively investigated in animal models. Murine p53 can directly repress the activation of IL-6 promoter [25]. In addition, p53 inhibits the transcription of TNF-inducible genes and NF-κB-dependent promoters and, consistently, p53 deficiency in macrophages or mast cells enhances the production of proinflammatory cytokines such as IL-1, IL-6, IL-12, and TNF-α [26]. Furthermore, Kawashima et al. [27] have found that p53 enhances the transcription of *Foxp3* gene and induces the differentiation of T regulatory cells, deficiency of which causes systemic autoimmune diseases. Moreover, *p53*^−/−^ mice treated with low-dose streptozotocin showed a higher rate of T1D incidence and higher levels of proinflammatory cytokines [28]. Consistent with P53 putative regulatory effect in autoimmunity, the presence of anti-P53 antibodies has been described in patients with some autoimmune disorders [29] including T1D [30]. Furthermore, upregulated TP53 pathways and increased susceptibility to apoptosis of CD4^+^CD25^high^ T regulatory cells have been observed in T1D [31].

Some studies have investigated the associations between *TP53* codon 72 polymorphism and susceptibility to inflammatory and autoimmune diseases. The *TP53* codon 72 Arg/Arg polymorphism has been associated with higher risk of inflammatory bowel disease [32]. On the other hand, Pro/Pro homozygotes demonstrate increased risk of endometriosis [33] and the Pro allele may be involved in the development of coronary artery disease [34]. Ruggeri et al. [35] and Chen at al. [36] showed an increased prevalence of the homozygous genotype Arg/Arg in Hashimoto’s thyroiditis patients. We obtained some conflicting results. In our study, we observed that the Arg/Arg variant is associated with decreased risk of autoimmune thyroiditis. The main reason for this discrepancy may be the fact that our patients had T1D and TA, not only Hashimoto’s thyroiditis. Moreover, population age distribution was different in our cohort and it is known that age of Hashimoto’s thyroiditis onset may influence other autoimmune disease clustering [37].

Diabetes is often associated with dysregulation of lipid metabolism and subsequently dyslipidemia. In the present study, we have observed that the Pro/Pro genotype and Pro allele are associated with an increased risk of dyslipidemia. Moreover, we have found that Pro/Pro homozygotes and Pro carriers had the highest concentrations of total and LDL cholesterol. Mounting evidence suggests that P53 plays a crucial role in normal as well as disturbed lipid metabolism [38]. Therefore, one may speculate that *TP53* codon 72 polymorphism constitutes an additional modulator of this process.

Some previous findings suggested that Pro/Pro carriers are more privileged during events contributing to inflammation and metabolic homeostasis, probably due to better retention of correct setting of inflammatory and metabolic parameters even in the presence of severe disturbance [39]. As the Arg variant is a stronger apoptosis inducer while the Pro variant is a stronger transcriptional activator, tissue-specific differences between these phenotypes have been observed [40]. While unraveling this issue, we have to bear in mind that, as for many transcription factors, the overall effect of the protein comes from its expression in leukocytes as well as non-immune cells such as the pancreatic islets. Moreover, effector T cell dysregulation is observed upon *p53* activation in T1D patients [41]. Intriguingly, multiple studies have shown that the *TP53* codon 72 polymorphism can affect apoptosis in the context of not only the wild-type *TP53* sequence but also the *TP53* with sustained somatic mutations. Interestingly, some reports suggest the association of *TP53* codon72 Pro isoform with higher levels of apoptosis [42]. Furthermore, there are still many unmeasured genetic and environmental factors that may modulate the effect of P53 on cell fates and as such, can contribute to the T1D complication and comorbidity development.

Thus, although further studies are required, the *TP53* codon 72 polymorphism could be considered a genetic marker to predict increased susceptibility to some T1D complications and comorbidities.

The present study has strengths and limitations that need to be briefly addressed. The advantages include the use of a pure Caucasoid population from the north region of Poland to eliminate false positive results due to population stratification. Regarding limitations, although the study cohort is homogenous and well-characterized, it may be considered relatively small. Therefore, conducting further studies on a larger group, especially with the consideration of its genetic substructure, is needed to confirm our results. In spite of these limitations, our findings emphasize the role of the *TP53* codon 72 polymorphism in T1D complications and comorbidities.

## Data Availability

All data generated or analyzed during this study are included in this published article.
